# Compound Specific Extraction of Camptothecin from *Nothapodytes nimmoniana* and Piperine from *Piper nigrum* Using Accelerated Solvent Extractor

**DOI:** 10.1155/2014/932036

**Published:** 2014-01-02

**Authors:** Vinayak Upadhya, Sandeep R. Pai, Ajay K. Sharma, Harsha V. Hegde, Sanjiva D. Kholkute, Rajesh K. Joshi

**Affiliations:** ^1^Regional Medical Research Centre, Indian Council of Medical Research (ICMR), Nehru Nagar, Belgaum, Karnataka 590 010, India; ^2^Department of Pharmacy, G.S.V.M. Medical College Kanpur, Uttar Pradesh 208002, India

## Abstract

Effects of varying temperatures with constant pressure of solvent on extraction efficiency of two chemically different alkaloids were studied. Camptothecin (CPT) from stem of *Nothapodytes nimmoniana* (Grah.) Mabb. and piperine from the fruits of *Piper nigrum* L. were extracted using Accelerated Solvent Extractor (ASE). Three cycles of extraction for a particular sample cell at a given temperature assured complete extraction. CPT and piperine were determined and quantified by using a simple and efficient UFLC-PDA (245 and 343 nm) method. Temperature increased efficiency of extraction to yield higher amount of CPT, whereas temperature had diminutive effect on yield of piperine. Maximum yield for CPT was achieved at 80°C and for piperine at 40°C. Thus, the study determines compound specific extraction of CPT from *N. nimmoniana* and piperine from *P. nigrum* using ASE method. The present study indicates the use of this method for simple, fast, and accurate extraction of the compound of interest.

## 1. Introduction

**Figure 1:**
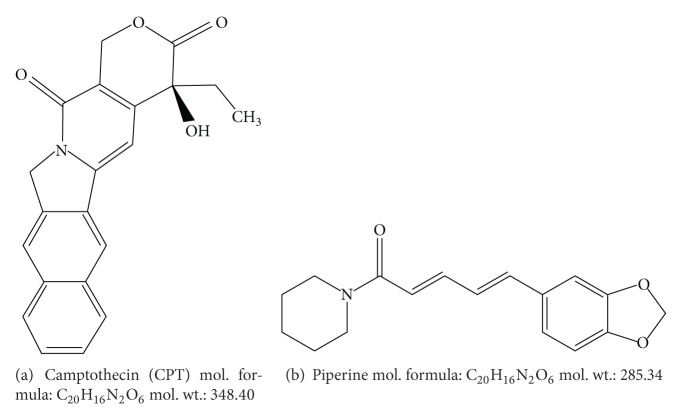
Chemical structures of camptothecin and piperine.

Camptothecin (CPT) a known potent anticancer active compound and piperine an economically important high valued alkaloid were used as the marker compounds (Figure 1(a)). Camptothecin was originally isolated from a Chinese tree *Camptotheca acuminata* (Nyssaceae) [1]. It is also reported in *Nothapodytes nimmoniana* and few other species belonging to unrelated orders of angiosperm classification [2–4]. *Nothapodytes nimmoniana* occupies important position in plant-based anticancer drugs because of CPT. Enormous demand for this alkaloid worldwide in the recent years has been subject to haphazard exploitation of the populations from wild. More than 20% decline in the population of *N. nimmoniana* from Western Ghats region has led to classify it in “*vulnerable*” category [5].

Piperine, an important alkaloid, has been reported from the fruits of many wild species and domesticated cultivars of *Piper nigrum* L. (Figure 1(b)) [6–8]. *Piper nigrum* also known as “King of Spices” (black pepper) is considered an important commodity of commerce in agriculture [9].

Identification and quantification of metabolites by any analytical technique depend upon its extraction. Extraction may refer to separation of analytes from a complex matrix. The extraction efficiency is greatly influenced by factors such as: solvent composition, solvent to solid ratio, temperature, time, and method of extraction [10–12]. Till date the number of extraction methods has been implied for extraction of CPT [13, 14] and piperine [15–17] by using Soxhlet, continuous shaking, ultrasonication, microwave assisted extractions, and many more. However, most of the methods consume both time and solvents and very few are effective in complete extraction of analytes or compounds of interests.

A thorough learning and understanding of the experimental optimization is essential for validation and commercial application of the process. Accelerated Solvent Extractor (ASE) is a new technique applied to extract organic compounds from a variety of samples to optimize solvent condition and to reduce extraction time. It accelerates the extraction process by elevating temperature at high pressure of the solvents. Therefore, important plant metabolites and their optimization of extraction method are the need of today. Thus the present work implies optimization of extraction method using ASE and studies the effect of temperature on extraction efficiency of CPT and piperine from *N. nimmoniana* and *P. nigrum*, respectively.

## 2. Materials and Methods

### 2.1. Chemical Reagents and Standards

Standards camptothecin and piperine (HPLC grade) were obtained from Sigma-Aldrich (India). HPLC grade acetonitrile, methanol, ethanol, glacial acetic acid, and water were used for analysis.

### 2.2. Collection and Preparation of Plant Material

Stem parts of *N. nimmoniana* and fruits of *P. nigrum* were collected from Belgaum (N 15.6383° E 074.2784°) and North Canara (N 14.4721°, E 074.5131°), region of Western Ghats of Karnataka, India. Herbaria of plant twigs were authenticated and deposited at Regional Medical Research Centre (RMRC), Indian Council of Medical Research (ICMR), Belgaum, Karnataka, India, for future reference (Voucher Numbers-*N. nimmoniana*: RMRC 1313 and* P. nigrum*: RMRC 1213). The plant materials were dried at room temperature and grounded to powder. The powdered material was sieved through a 20 *μ*m stainless sieve and taken for further analysis.

### 2.3. Accelerated Solvent Extractor (ASE) Sample Preparation

Extraction was carried out in Accelerated Solvent Extraction system ASE 350 (Dionex Corporation, Sunnyvale, CA, USA) equipped with a solvent controller unit. The cells of 5 mL capacity were employed for the study. Two cellulose filters were placed at the bottom of the sample cells before filling. The sample cells were filled with 1 g dried stem powder of *N. nimmoniana* and 0.1 g dry fruit powder of *P. nigrum* was utilized for extraction separately. Three scoops (~2.5 g) of ASE prep diatomaceous earth (Dinoex Corporation, Sunnyvale, California) were mixed with plant powder and loaded on the cell tray. Methanol and ethanol were used for extraction of CPT and piperine, respectively. The selection of the solvents for extraction was based on earlier reports [13–15]. To assure complete extraction, a particular sample cell at a given temperature was extracted in 3 cycles.

### 2.4. Quantification of Camptothecin and Piperine Using Reversed Phase-Ultraflow Liquid Chromatographic (RP-UFLC) Analysis

#### 2.4.1. Instrumentation

The reversed phase-ultraflow liquid chromatographic (RP-UFLC) analysis was performed on Shimadzu chromatographic system (Model number LC-20AD) consisting of a quaternary pump, manual injector, degasser (DGU-20A5), and dual *λ* UV absorbance diode array detector SPD-M20A. The built-in LC-Solution software system was used for data processing. Chromatographic separation was achieved on a Hibar RP-select B column (LiChrospher 60, 5 *μ*m, 4.6 × 250 mm) for CPT and RP-18e (LiChrospher 100, 5 *μ*m, 4.6 × 250 mm) for piperine.

#### 2.4.2. Chromatographic Conditions

Mobile phase consisting of acetonitrile : water (40 : 60) for CPT and methanol : water (70 : 30) for piperine was used for separation with an injection volume of 20 *μ*L. A chromatographic condition of 1.0 and 1.4 mL min^−1^ flow rate at 254 and 343 nm was set for CPT and piperine, respectively. The retention time was observed 8 min for CPT and 10 min for piperine.

#### 2.4.3. Calculations, Calibration Curves, and Linearity

Camptothecin was accurately weighed and dissolved in few drops (50 *μ*L) of DMSO by warming and the volume was made with methanol to produce a standard stock solution (0.5 mg mL^−1^). Similarly, 1 mg mL^−1^ stock solution of piperine in methanol was prepared. The stock solutions of CPT and piperine were prepared and serially diluted with respective solvents to obtain working concentrations for plotting calibration curves. Seven different concentration levels of CPT (0.001, 0.01, 10, 20, 40, 80, and 100 *μ*g mL^−1^) and nine of piperine (0.01, 1, 3, 10, 50, 100, 200, 400, and 600 *μ*g mL^−1^) were used for the study. All the solutions and analytes were stored in microfuge tubes at 4°C until further use.

#### 2.4.4. System Suitability, LOD, and LOQ

The system suitability test was assessed by three replicates of standard CPT and piperine at a particular concentration 40 and 50 *μ*g mL^−1^, respectively. The peak areas were used to evaluate repeatability of the method and analyzed for resolution and tailing factors. The limit of detection (LOD) and limit of quantification (LOQ) were determined with the signal : noise method. Signal : noise ratios of 3.3 and 10 were used for estimating the LOD and LOQ, respectively.

## 3. Results and Discussions

The present study signifies use of improved, simple, fast, and accurate method of extraction by using ASE. The work was carried out to study the effect of varying temperatures at a constant pressure for determination of two chemically different but pharmacologically and commercially important compounds (CPT and piperine). Both the compounds were analyzed on reverse phase columns under isocratic system as described in experimental section.

**Figure 2:**
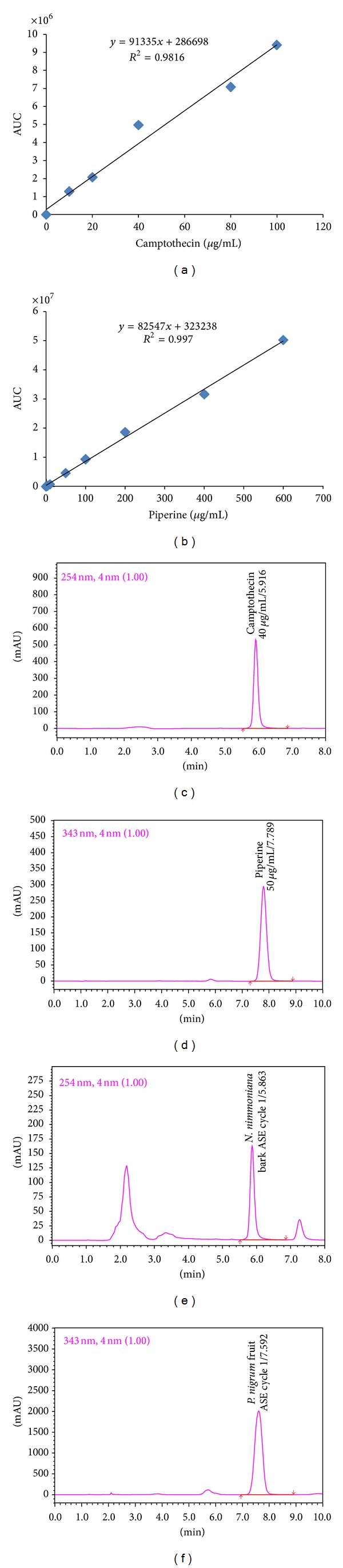
(a) Calibration curve of camptothecin; (b) calibration curve of piperine; (c) standard camptothecin (40 *μ*g mL^−1^); (d) standard piperine (50 *μ*g mL^−1^); (e) camptothecin from stem of *N. nimmoniana* extracted by ASE (Cycle 1); (f) piperine from fruits of *P. nigrum *extracted by ASE (Cycle 1).

Quantitative determination of CPT and piperine were achieved using RP-UFLC method and the results were expressed as g 100 g^−1^ on dry weight basis. Calibration curves were constructed against their area under curve to obtain a regression equation with coefficients of determination (*R* ^2^) above 0.980 (Figures 2(a) and 2(b)). This was used to estimate CPT and piperine content from both species. To reduce the impurity matrix and for quantification within the range of standard concentrations, a 1 : 9 dilution for extracts of *N. nimmoniana *was made.  Table 1 represents conditions for ASE method and details of UFLC analysis. The lowest concentrations were 0.001 (CPT) and 0.01 *μ*g mL^−1^ (piperine) for calibration. The relative standard deviation (RSD) values for both analytes that were found less than 2% indicate that the methods used in this study were précised and reproducible. Validation of the method was carried out by spiking known amount of CPT and piperine standards to equal volume of sample extracts to obtain recovery within the range of 95–100% for both.

**Figure 3:**
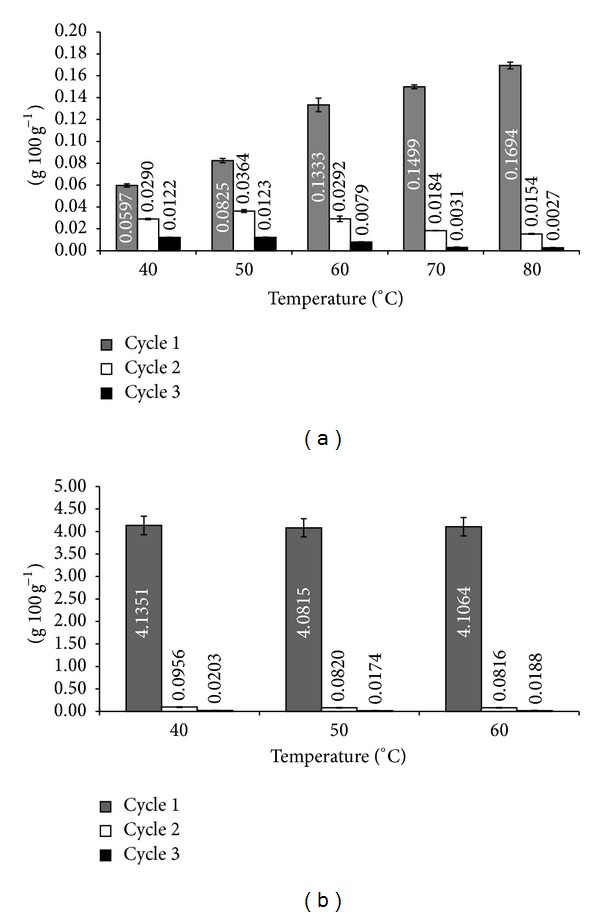
Content yield of Accelerated Solvent Extractor (ASE): (a) camptothecin (g 100 g^−1^) in stem extracts of *N. nimmoniana*; (b) piperine (g 100 g^−1^) in fruit extracts of *P. nigrum*.

Profiles with retention time of 5.9326 ± 0.051 min for CPT and 7.7694 ± 0.0900 min for piperine in standards and samples were obtained as final output (Figures 2(c)–2(f)). Clear, sharp peaks of standard compounds ensured purity (98%) and also reduced compatibility issues between extractive solvents and mobile phase in the analysis. The autoscaled chromatograms were generated for 3 cycles each for *N. nimmoniana* and *P. nigrum* extracts at varying temperatures (Figures 2(e), 2(f), 3(a), and 3(b)). All samples were detected above LOD and quantifications above LOQ (Table 1) for CPT and piperine.

A constant increase in the content of CPT after step wise (10°C) increase in temperature up to 80°C was observed at cycle 1, whereas successive cycles 2 and 3 showed decline in CPT content (Figure 3(a)). Highest content of CPT in *N. nimmoniana* stem extract was observed in cycle 1 at maximum temperature elevation of 80°C (Figures 2(e) and 3(a)). It is well understood that temperature is a significant factor influencing the rate of extraction. Thus, the relationship between rate of extraction and temperature is important in designing extraction methods of plant-based materials [13]. The total CPT content taken as a sum of cycle 1, 2, and 3 was 0.1875 g 100 g^−1^ dry weight. The yield of CPT in this study was found comparatively higher than the earlier reports [3, 18]. On contrary, minute variation was observed in piperine content from fruits of *P. nigrum* in all the cycles studied using ASE (Figure 3(b)). Cycle 1 at each step of temperature (40, 50 and 60°C) yielded a mean highest amount of piperine (4.1077 ± 0.0268 g 100 g^−1^ dry weight) compared to cycle 2 (0.0864 ± 0.0079 g 100 g^−1^ dry weight) and cycle 3 (0.0188 ± 0.0014 g 100 g^−1^ dry weight), indicating diminutive effect of temperature and pressure on extraction yield of piperine. The total piperine content (cycle 1 + 2 + 3) in our finding was found in the range of earlier reports [19, 20].

The time required for completion of each extraction stage was 6.5 min including purge, heat, and static time. ASE proves to be the better option in studying temperature-dependent extractions of compounds from plant based matrices. The temperature-based extractions not only reduced time but also are simple, fast, and accurate. The extraction method was found efficient with small amount of plant material (0.1 g dried fruit powder of *P. nigrum*), signifying, its utility in standardization and quality control of herbal medicines. Besides, use of parameters such as pressure, time, and choice of solvents makes more appropriate extraction of plant materials. This study also proposes compound specific extraction by using ASE and its suitability for obtaining optimum yield of compound of interest with less solvent consumption and time.

## 4. Conclusions

Results of the study conclusively affirm that compound-based extraction by using ASE from complex plant matrices is a suitable tool for standardization or quality control of the herbal products. The study indicated simple, fast, and accurate extraction methods for extraction of the compounds from different plant materials. Significant variation in the method of extraction for both the compounds was noticed. Therefore, these new improved and automated extraction methods would complement for extraction-based experiments.

## Figures and Tables

**Table 1:** Accelerated Solvent Extraction (ASE) conditions for extraction and UFLC attributes during determination of CPT and piperine from *N. nimmoniana* and *P. nigrum*, respectively.

Plant name	*N. nimmoniana *	*P. nigrum *
Compound	Camptothecin	Piperine
Sample size (g)	1.0	0.1
Extraction solvents	Methanol	Ethanol
Temperature range (°C)	40–80	40–60
Temperature elevation (°C)	10	10
Heat (min)	5	5
Max pressure range (psi)	1525–1675	1520–1570
Static time (min)	1.0	1.0
Static cycles	1.0	1.0
Flush (%)	10	10
Purge time (min)	0.5	0.5
Total extraction time/cycle (min)	6.5	6.5
Linearity equation	*y* = 81036*x* + 41525	*y* = 91335*x* + 28669
*R* ^2^	0.992	0.981
LOD (*µ*g mL^−1^)	0.2020	0.1173
LOQ (*µ*g mL^−1^)	0.6121	0.3554
